# The Interplay between Inflammation and Fibrosis in Kidney Transplantation

**DOI:** 10.1155/2014/750602

**Published:** 2014-06-04

**Authors:** Irina B. Torres, Francesc Moreso, Eduard Sarró, Anna Meseguer, Daniel Serón

**Affiliations:** ^1^Nephrology Department, Vall d'Hebron University Hospital, Autonomous University of Barcelona, Passeig Vall d'Hebron 119-129, 08035 Barcelona, Spain; ^2^Renal Physiopathology Department, CIBBIM-Nanomedicine, Vall d'Hebron Research Institute, Passeig Vall d'Hebron 119-129, 08035 Barcelona, Spain

## Abstract

Serial surveillance renal allograft biopsies have shown that early subclinical inflammation constitutes a risk factor for the development of interstitial fibrosis. More recently, it has been observed that persistent inflammation is also associated with fibrosis progression and chronic humoral rejection, two histological conditions associated with poor allograft survival. Treatment of subclinical inflammation with steroid boluses prevents progression of fibrosis and preserves renal function in patients treated with a cyclosporine-based regimen. Subclinical inflammation has been reduced after the introduction of tacrolimus based regimens, and it has been shown that immunosuppressive schedules that are effective in preventing acute rejection and subclinical inflammation may prevent the progression of fibrosis and chronic humoral rejection. On the other hand, minimization protocols are associated with progression of fibrosis, and noncompliance with the immunosuppressive regime constitutes a major risk factor for chronic humoral rejection. Thus, adequate immunosuppressive treatment, avoiding minimization strategies and reinforcing educational actions to prevent noncompliance, is at present an effective approach to combat the progression of fibrosis.

## 1. Introduction

**Figure 1:**
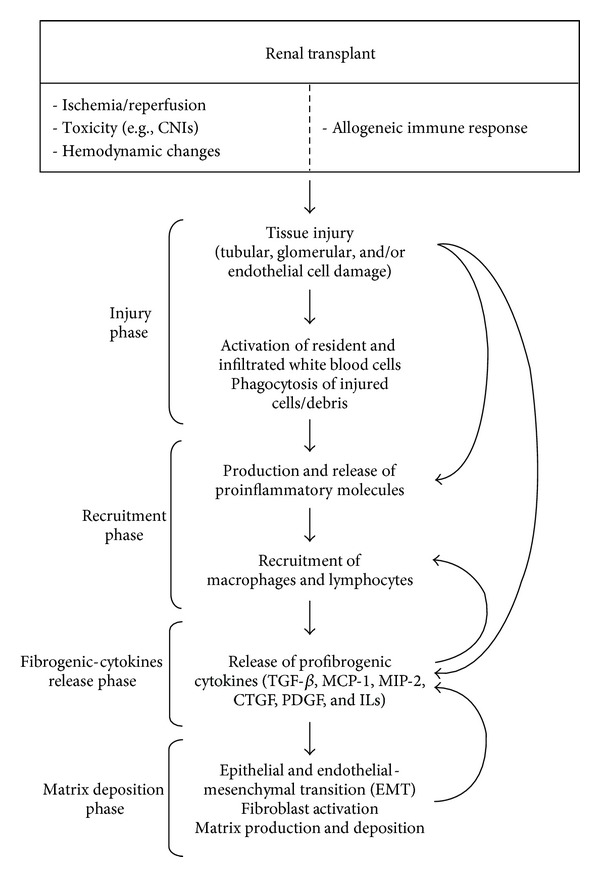
Renal transplant-induced fibrosis involves a complex multifactorial inflammatory process with the participation and interaction of infiltrated cells with different cell types in the kidney and is orchestrated by a network of cytokines/chemokines, growth factors, adhesion molecules, and signalling processes. These events include several phases in a dynamic process in which many of these events occur simultaneously, often in a mutually stimulating fashion.

Progressive renal fibrosis, regardless of the underlying aetiology, is the final common manifestation of a wide variety of chronic kidney diseases (CKD) that lead to end-stage renal disease. Fibrosis is a process of normal wound healing and repair that is activated in response to injury to maintain the original tissue architecture and functional integrity. However, prolonged chronic injurious stimuli may cause deregulation of normal processes and result in an excess deposition of extracellular matrix (ECM) [1]. Continuous deposition of ECM results in fibrous scars and distorts the architecture of kidney tissues, leading to the collapse of renal parenchyma and the loss of kidney function [2]. Chronic injury involves a complex multistage inflammatory process with inflammatory cell infiltration, mesangial and fibroblast activation, tubular-epithelial to mesenchymal transition, endothelial to mesenchymal transition, cell apoptosis, and extracellular matrix expansion that is orchestrated by a network of cytokines/chemokines, growth factors, adhesion molecules, and signalling processes [3, 4]. These events include several phases summarized in Figure 1: (i) tissue injury and activation, (ii) recruitment of inflammatory cells, (iii) release of fibrogenic cytokines, and (iv) activation of collagen-producing cells. However, it should be stressed that renal fibrogenesis is a dynamic process in which many of these events occur simultaneously, often in a mutually stimulating fashion [2]. The injury phase, which can be induced by a variety of noxious stimuli including immunological, metabolic, hemodynamic, ischemic, and toxic assaults, results in the production and release of proinflammatory molecules caused by cytokine-mediated endocytosis/phagocytosis [5–8]. Neutrophils are the first cells recruited, as they uptake cell debris and phagocytose apoptotic bodies facilitating the repair of the lost tissue components, resulting in a reconstitution of the original tissue architecture and function. This beneficial repairing process can be detrimental when proceeding in an uncontrolled manner, then leading to progressive fibrosis with a loss of function [9]. Thus, controlling excessive inflammation would be of great potential therapeutic benefit for inhibiting progressive fibrosis of kidney.

## 2. Molecular Mechanisms Leading to Fibrosis Progression

The pathogenesis of inflammation is complex and multifactorial, involving the interaction of cytokines, chemokines, and adhesion molecules. The participation and interaction of infiltrated cells with different cell types in the kidney is required to promote renal fibrosis. Depending on the aetiology of renal injury, tubular, glomerular, or interstitial infiltrated inflammatory cells become activated and produce fibrogenic and inflammatory cytokines. Inflammatory infiltrates, including neutrophils, macrophages, and lymphocytes, are evident in experimental models of renal disease and human renal biopsy specimens [10]. Activation of peritubular capillary endothelial cells may facilitate the recruitment of interstitial mononuclear cells. Following neutrophils, macrophages infiltrate damaged tissues and phagocytose and secrete fibrogenic cytokines. Macrophages are a major source of transforming growth factor-*β* 1 (TGF-*β*1) in fibrosing organs. T and B lymphocytes are also recruited to the site of injury and further facilitate secretion of fibrogenic cytokines [11]. At the same time, TGF-*β*1 is a potent chemoattractant for cells of macrophage-monocytic lineage. In addition to TGF-*β*1, monocyte chemoattractant protein-1 (MCP-1), macrophage inflammatory protein-1 (MIP-1), and macrophage inflammatory protein-2 (MIP-2) are also involved in recruitment of inflammatory cells [12]. The gradients of chemoattractant cytokines released by damaged tubular cells provide a directional signal for guiding the infiltration of inflammatory monocytes/macrophages and T cells to the injured sites and are thought to play an important role in this inflammatory process.

Members of the TGF-*β* superfamily are the most extensively studied growth factors that have been linked to renal fibrosis [13]. Macrophages, tubular epithelial cells, and myofibroblasts are all capable of synthesizing TGF-*β* at different stages during the development of renal fibrotic lesions [14]. However, the observation that macrophage ablation markedly attenuates fibrosis in various conditions suggests that these cells are among the main producers of this growth factor [15, 16]. Macrophages are heterogeneous and can be classified by distinct phenotypic markers that correspond to different subsets with distinct functional capabilities, including important roles in tissue repair and remodelling [17].

Although different fibrogenic factors have been documented, including various cytokines and hormonal, metabolic, and hemodynamic factors, it is widely accepted that TGF-*β* and its downstream Smad signalling play an essential role. Upregulation of TGF-*β* is a universal finding in virtually every type of CKD, both in animal models and in humans. Despite the well documented role of TGF-*β* in renal fibrosis, long-term inhibition of TGF-*β* action, in an attempt to hamper the progression of renal fibrosis, does not seem to be an optimal approach provided that TGF-*β* is also an anti-inflammatory cytokine. The profibrotic and anti-inflammatory properties of TGF-*β* pose a dilemma for the therapeutic application of TGF-*β* inhibition and this is one of the reasons that novel antifibrotic targets are under active investigation [18].

In renal fibrosis, the activation of the renin-angiotensin-aldosterone system and its main effector angiotensin II (AngII) stimulates vascular inflammation, upregulation of reactive oxygen species, cytokines, chemokines, and growth factors, and recruitment of infiltrating cells into the kidney [19, 20]. The relevance of AngII to renal fibrosis has immediate clinical relevance due to the availability of orally active inhibitory drugs. AngII has been shown to stimulate TGF-*β* production by various cells including renal tubular cells and fibroblasts and several studies have demonstrated that the use of either AngII receptor (AT1 and AT2) antagonists or angiotensin converting enzyme (ACE) inhibitors in experimental renal disease models reduces TGF-*β* production and attenuates renal interstitial fibrosis [21, 22].

In human kidney diseases, the activated renal renin-angiotensin system has been described. In diabetic nephropathy, elevated AngII generation did correlate with the presence of inflammatory cell infiltration, the activation of NF-*κ*B (nuclear factor kappa-light-chain-enhancer of activated B cells), and proinflammatory gene overexpression [23]. The inhibition of the NF-*κ*B pathway has also shown the prevention of inflammation in experimental renal damage. These observations emphasize the importance of treatments that block the AngII-induced inflammatory process in human renal diseases and provide a rationale to investigate further the involvement of the AT2/NF-*κ*B pathway in the inflammatory response in kidney diseases [20].

## 3. Molecular Mechanisms Leading to Fibrosis in Renal Transplantation

Inflammation has also been pinpointed as a hallmark for renal transplant functional decline. Inflammation, especially when it is associated with fibrosis in surveillance kidney biopsies, is a risk factor for long-term transplant failure. Park et al. [24] have shown that one-year surveillance biopsies with normal histology or fibrosis had stable renal function between 1 and 5 years, whereas those with both fibrosis and inflammation exhibited a decline in GFR and reduced graft survival. Immunohistochemistry confirmed increased interstitial T cells and macrophages/dendritic cells in the group with both fibrosis and inflammation, and there was increased expression of transcripts related to innate and cognate immunity. These authors demonstrated elevated expression of multiple innate and adaptive immune mediators consistent with tissue injury response, Th1-type T cell response, and suppression of counterregulatory pathways. Microarray analyses confirmed and extended this profile, revealing the overexpressed pathways and gene clusters in the interstitial fibrosis and inflammation group to be heavily enriched for immune activation and identifying the process as being closely linked with IFN-*γ*-induced, cytotoxic T lymphocyte-associated, and acute rejection signatures. Pathway analysis of microarray data for the interstitial fibrosis and inflammation group also provided evidence of active participation of a range of immunologic cell types, including T cells, B cells, monocyte/macrophages, dendritic cells, and natural killer cells. Results of this study indicate that early surveillance histology with or without targeted molecular analysis provides important prognostic information. It has been suggested that analysis of intragraft innate and adaptive immune pathways during early posttransplantation years may provide the basis for early interventions aimed at altering rejection-like inflammation improving long-term survival of kidney allografts [24–26].

## 4. Preexisting Kidney Fibrosis and Graft Outcome

Despite the fact that the use of new immunosuppressants has allowed reduction in the incidence of acute rejection and an improvement of short-term results in renal transplantation, long-term graft survival has been only marginally increased [27]. Among immune and nonimmune mechanisms influencing graft survival, donor related factors are one of the major determinants of graft outcome [28, 29].

The increased utilization of the so-called expanded criteria donors during the last years, that is, donors older than 60 years or donors older than 50 years with two of the following conditions, death due to stroke and history of hypertension and serum creatinine > 1.5 mg/dL, implies that a high proportion of kidneys already display interstitial fibrosis, tubular atrophy, vascular intimal thickening, and glomerulosclerosis at the time of transplantation. The severity of these lesions is associated with delayed graft function, decreased glomerular filtration rate, and decreased allograft survival. Accordingly, different scores to evaluate the severity of preexisting damage have been proposed [30–32]. Although intra- and interobserver reproducibility of these measures is not ideal, the majority of studies have shown a close association between the severity of fibrosis and graft outcome [33, 34]. It has been proposed that the modest improvement on long-term allograft survival despite decreased incidence of acute rejection with actual immunosuppression is mainly explained by the increased use of kidneys already displaying fibrosis.

## 5. Ischemia/Reperfusion Injury and Kidney Fibrosis

Ischemia/reperfusion injury (IRI) is a key event in organ transplantation since restoration of blood flow to ischemic tissue exacerbates tissue damage by initiating a cascade of inflammatory events including release of proinflammatory cytokines and chemokines, recruitment of leukocytes, and activation of the complement system [35]. Different experimental and clinical studies have shown that transplant IRI may impact short- and long-term graft survival following kidney transplantation and is strongly associated with delayed graft function [36]. Acute kidney injury is associated with an extensive loss of the corticomedullary proximal tubular epithelial cells and with a reduction in the number of peritubular capillaries [37]. Moreover, delayed graft function increases the immunogenicity of the allograft and the risk of acute rejection episodes [36]. The initiation of profibrotic pathways is also relevant as shown by the increased expression of TGF-*β* and activation of NF-*κ*B in allografts that developed chronic changes subsequent to the occurrence of acute tubular injury [38]. These phenomena, inherently present in the majority of the grafts, can be more pronounced in expanded criteria donors since these allografts have a limited capacity to repair parenchymal damage and could exhaust the ability of tubular epithelial cells to regenerate. Additionally, these processes could lead to accelerated senescence and aggravate the progression of interstitial fibrosis and tubular atrophy [39].

## 6. Fibrosis Progression in Surveillance Biopsies

Preexisting chronic donor damage can progress after transplantation due to the different immunologic and nonimmunologic insults to which the kidney is exposed. To evaluate the progression of fibrosis after transplantation different groups have performed surveillance biopsies at different time points after transplantation. From the initial reports, it became clear that chronic histological damage in the tubule-interstitial, vascular, and glomerular compartments rapidly progresses during the initial months after transplantation while renal function remains stable. In different studies it has been shown that the presence of interstitial fibrosis/tubular atrophy (IF/TA) involved about 40% of transplants at 3–6 months [39, 40], 50% at 1 year [41], and 65% at 2 years [42]. The progression of IF/TA was associated with an increased incidence of acute rejection before performing the surveillance biopsy and with a lower immunosuppressive treatment. Furthermore, it has been consistently shown that the presence of IF/TA adjusted for renal function at the time of biopsy is closely associated with long-term graft survival. However, since IF/TA is a nonspecific lesion that can be related with different immune and nonimmune injuries to the graft, during the last years a big effort has been done to characterize causes of late graft failure. In these studies, it has been shown that specific disease entities may be identified in more than 90% of cases, antibody-mediated rejection and glomerular disease being the leading causes of late graft failure [43, 44]. Recently, to integrate this apparent discrepancy, it has been shown that early chronic histological damage was an independent risk factor for late graft loss, irrespective of whether a specific, progressive disease was diagnosed or not [45]. Thus, the burden of fibrosis modulates outcome in different renal allograft diseases.

## 7. Inflammation as a Risk Factor for Progression of Kidney Fibrosis

**Figure 2:**
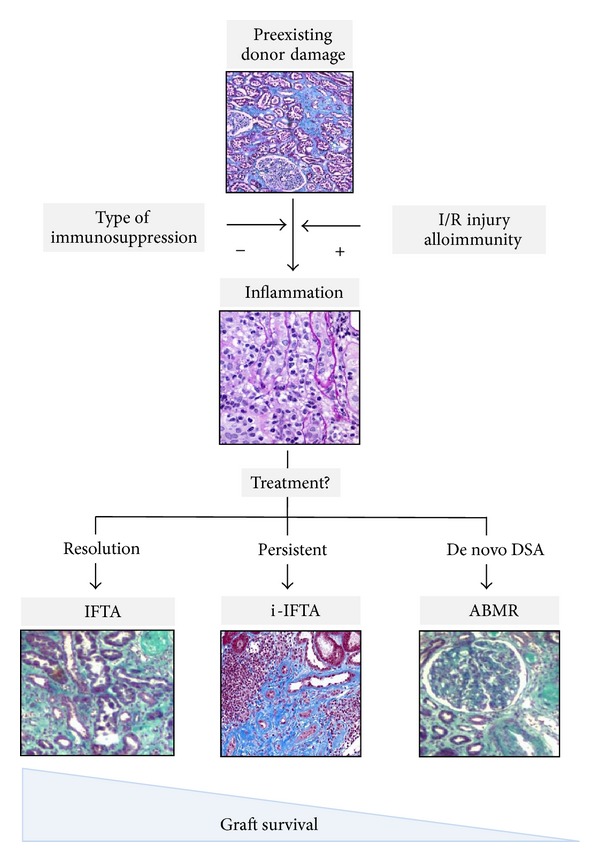
Progression of fibrosis after kidney transplantation. Fibrosis is already present in a proportion of grafts, especially in renal allograft obtained from expanded criteria donors. Ischemia/reperfusion (I/R) injury and alloimmune response trigger inflammation and its severity is modulated by immunosuppressive treatment. Subclinical inflammation can be ameliorated by treatment with steroid boluses or by increasing exposure to immunosuppressive drugs. Quiescent interstitial fibrosis/tubular atrophy (IF/TA) may represent the healing of the inflammatory insult while inflammation in areas of fibrosis (i-F/TA) and antibody-mediated rejection (ABMR) due to the appearance of de novo donor specific antibodies (DSA) may represent an ongoing inflammatory response that is associated with decreased allograft survival.

The largest study contributing to describing the natural history of the evolution of inflammation and chronic damage in stable grafts was conducted on 120 recipients receiving simultaneous kidney-pancreas transplantation in whom near 1.000 surveillance biopsies were done during 10 years of follow-up. Most severe inflammation was already observed during the first months after transplantation and tended to decrease during the first year although the inflammation persists after the first year in a proportion of patients. At the same time, interstitial fibrosis rapidly progressed during the first months after transplant. Beyond one year, glomerulosclerosis and intimal thickening slowly progressed as well as the severity of IF/TA. The presence of severe chronic lesions was associated with declining renal function and graft failure [46]. It has been shown that early inflammation observed in surveillance biopsies is associated with the progression of IF/TA [47, 48] and with decreased renal allograft survival [49]. However, the classification of surveillance biopsies as (i) normal histology, (ii) fibrosis without inflammation, (iii) inflammation without fibrosis, and (iv) inflammation associated with fibrosis leads to the observation that only patients with inflammation associated with fibrosis showed a decreased renal allograft survival [24, 50]. Additionally, it has been shown that the presence of interstitial inflammation in areas of fibrosis (i-IFTA) in diagnostic biopsies is especially harmful for the graft [51]. Studies conducted on sequential biopsies have shown that acute cellular rejection, BK nephropathy, increasing number of HLA mismatches, retransplantation, and delayed graft function were risk factors for the presence of i-IFTA in one-year surveillance biopsies [48, 52]. More recently, it has been described that early inflammation after transplantation evaluated by means of surveillance biopsies is associated with an increased risk to develop de novo donor HLA specific antibodies and chronic antibody-mediated rejection [53–55]. In summary, early inflammation is associated with three different conditions, interstitial fibrosis, interstitial fibrosis associated with inflammation, and chronic allograft rejection. However, graft survival is shortened in patients with i-IF/TA and chronic humoral rejection in comparison to patients with quiescent fibrosis. In Figure 2, the relationship between events modulating early inflammation after transplant and late different histological phenotypes is shown.

## 8. Treatment of Subclinical Rejection to Slow Kidney Fibrosis Progression

Since subclinical inflammation is indistinguishable from inflammation observed in episodes of acute cellular rejection, it was tempting to propose that treatment of subclinical inflammation with steroid boluses may improve outcome after renal transplantation. The first to test this hypothesis was Rush et al. [56] in an elegant prospective randomized clinical trial in which patients were randomized to be biopsied at 1, 2, and 3 months and treated with steroid boluses in case they showed subclinical inflammation. The control group was not biopsied at these time points and, accordingly, not treated for subclinical inflammation. Fibrosis at 6 months was less severe in patients that were biopsied and treated for subclinical inflammation. This was the first study to show, as a proof of concept, that treatment of subclinical inflammation prevents progression of fibrosis. It is important to remark that patients enrolled in this study were treated with cyclosporine, azathioprine, and prednisone, a regimen associated with a high prevalence of acute rejection and subclinical inflammation. In this study, over 50% of patients showed subclinical inflammation at the time of surveillance biopsy. A similar study was done more recently [57] in which patients were randomized to be biopsied at 1 and 4 months and treated with steroid boluses in case they presented subclinical inflammation. The control group was again not biopsied and accordingly not treated. Baseline immunosuppression consisted in a cyclosporine or tacrolimus based regimen. The prevalence of subclinical inflammation was 39% at 1 month and 26% at 4 months, a lower figure than in the previous study. Estimated glomerular filtration rate at 6 months and 1 year was better in patients that were biopsied and treated, suggesting that treatment of subclinical inflammation was associated with preservation of renal function.

Rush et al. published in 2007 the results of a multicentre trial in which patients treated with tacrolimus, mycophenolate, and prednisone were randomized, as in his previous study, to be biopsied at 1, 2, and 3 months and treated with steroid boluses in case they presented subclinical inflammation. The control group was again not biopsied and, accordingly, not treated. There were no differences between groups in the progression of fibrosis evaluated by means of a 6- and 24-month surveillance biopsy and the evolution of renal function was also not different between groups. Most remarkably, overall incidence of subclinical inflammation was less than 10% at 1, 2, and 3 months, suggesting that treatment with tacrolimus, mycophenolate mofetil, and prednisone may efficiently prevent early inflammation [58]. After this study the interest shifted from treatment to prevention of subclinical inflammation.

## 9. Prevention of Subclinical Inflammation to Avoid Kidney Fibrosis

The prevalence of subclinical inflammation in three-month surveillance biopsies is lower in tacrolimus than in cyclosporine treated patients [59–61]. Quantification of the severity of inflammatory infiltrates with monoclonal antibodies confirmed that patients receiving tacrolimus showed less severe glomerular and interstitial inflammation than patients treated with cyclosporine [62]. These data suggested that the type of immunosuppressive treatment modulates the severity of inflammation after transplantation. Since inflammation is associated with progression of fibrosis, the question whether prevention of early inflammation by treatment may delay the progression of fibrosis was raised.

In a prospective trial in which patients were randomized to receive 4 different immunosuppressive schedules: cyclosporine associated with mycophenolate mofetil, tacrolimus associated with mycophenolate mofetil, cyclosporine associated with sirolimus, and tacrolimus associated with sirolimus, it was observed that regimens combining a calcineurin inhibitor with sirolimus showed a lower prevalence of acute rejection during the first year, a lower prevalence of subclinical inflammation at 1-year protocol biopsy, and less severe fibrosis evaluated by means of a surveillance biopsy at 5 years, suggesting that immunosuppressive schedules that are effective in preventing acute rejection and subclinical inflammation are also effective in preventing the progression of fibrosis [63]. At the time this paper was published, it was assumed that the combination of a calcineurin inhibitor and an inhibitor of the mammalian target of rapamycin (i-mTOR) was a nephrotoxic combination [64]. Thus, this study challenged the idea that avoidance of anticalcineurin treatments was the best strategy to prevent the progression of fibrosis [65]. In the Concept trial, patients receiving cyclosporine, mycophenolate mofetil, and prednisone were randomized to continue with the same schedule or to be switched from cyclosporine to sirolimus. At one year, the surveillance biopsy showed that the severity of fibrosis quantified by means of an image analysis technique was not different between groups [66], while the presence of subclinical inflammation was 45% in sirolimus and 15% in cyclosporine treated patients, suggesting that sirolimus is less effective in preventing inflammation than cyclosporine [67]. More recently, it has been shown that the early switch from cyclosporine to everolimus is associated with an increased risk of appearance of the novo HLA donor specific antibodies and chronic humoral rejection [68], reinforcing the notion that an i-mTOR based regimen may be less effective than a calcineurin inhibitor regimen to control the immune response after transplantation.

In the last two decades, the immunosuppressive schedule has changed from cyclosporine to tacrolimus based regimens. Thus, it is interesting to compare the prevalence of chronic lesions in surveillance biopsies obtained late after transplantation in these different periods, the cyclosporine and tacrolimus era. In 2003, in the paper published by Nankivell et al. [46], the prevalence of moderate or severe interstitial fibrosis at 5 years was 66% and in the Stegall et al. [69] paper published in 2011 it was 17%. Of note, hyaline changes were 90% in the first and 19% in the second study. Although such a comparison should be considered with caution, since patients characteristics between studies were different, it again suggests that the introduction of more powerful immunosuppressive schedules better controlling early inflammation may have changed the rate of progression of fibrosis after transplantation.

Further support for the role of immunosuppression in the prevention of early inflammation and progression of fibrosis comes from the observation that minimization of cyclosporine treatment was associated with progression of fibrosis when evaluated by means of 3- and 12-month surveillance biopsies [41]. Similarly, lower exposure to tacrolimus was also associated with accelerated progression of fibrosis evaluated again by means of surveillance biopsies done at 3 and 12 months. In this last study, low tacrolimus was also associated with higher prevalence of acute rejection, but high exposure to tacrolimus was not associated with lesions considered to represent anticalcineurin associated nephrotoxicity [70]. These results argue against minimization of immunosuppression, at least during the first months. Moreover, in the last years there is increasing evidence supporting a major role of patient's compliance in renal allograft survival [44, 71], and it has been also shown that patients enrolled in a special program aiming to improve treatment compliance have a better outcome than patients followed in the standard way [72].

## 10. Conclusions

Taken together, these data point out that inflammation early after transplantation is a major determinant of the progression of fibrosis, appearance of HLA donor specific antibodies, and graft outcome. On the other hand, an adequate immunosuppressive treatment, avoiding minimization strategies and reinforcing educational actions to prevent noncompliance, is at present an effective approach to combat the progression of fibrosis.
